# Classification of Cucumber Leaves Based on Nitrogen Content Using the Hyperspectral Imaging Technique and Majority Voting

**DOI:** 10.3390/plants10050898

**Published:** 2021-04-29

**Authors:** Sajad Sabzi, Razieh Pourdarbani, Mohammad Hossein Rohban, Alejandro Fuentes-Penna, José Luis Hernández-Hernández, Mario Hernández-Hernández

**Affiliations:** 1 Department of Biosystems Engineering, College of Agriculture, University of Mohaghegh Ardabili, Ardabil 56199-11367, Iran; s.sabzi@uma.ac.ir; 2 Computer Engineering Department, Sharif University of Technology, Tehran 14588-89694, Iran; 3 National Technological of México/Campus CIIDET, Querétaro 76000, Mexico; afuentes@ciidet.edu.mx; 4 National Technological of México/Campus Chilpancingo, Chilpancingo, Guerrero 39070, Mexico; joseluis.hernandez@itchilpancingo.edu.mx; 5 Faculty of Engineering, Autonomous University of Guerrero, Chilpancingo, Guerrero 39087, Mexico

**Keywords:** artificial neural network, cucumber, hyperspectral imaging, majority voting, nitrogen

## Abstract

Improper usage of nitrogen in cucumber cultivation causes nitrate accumulation in the fruit and results in food poisoning in humans; therefore, mandatory evaluation of food products becomes inevitable. Hyperspectral imaging has a very good ability to evaluate the quality of fruits and vegetables in a non-destructive manner. The goal of the present paper was to identify excess nitrogen in cucumber plants. To obtain a reliable result, the majority voting method was used, which takes into account the unanimity of five classifiers, namely, the hybrid artificial neural network–imperialism competitive algorithm (ANN-ICA), the hybrid artificial neural network–harmonic search (ANN-HS) algorithm, linear discrimination analysis (LDA), the radial basis function network (RBF), and the K-nearest-neighborhood (KNN). The wavelengths of 723, 781, and 901 nm were determined as optimal wavelengths using the hybrid artificial neural network–biogeography-based optimization (ANN-BBO) algorithm, and the performance of classifiers was investigated using the optimal spectrum. The results of a *t*-test showed that there was no significant difference in the precision of the algorithm when using the optimal wavelengths and wavelengths of the whole range. The correct classification rate of the classifiers ANN-ICA, ANN-HS, LDA, RBF, and KNN were 96.14%, 96.11%, 95.73%, 64.03%, and 95.24%, respectively. The correct classification rate of majority voting (MV) was 95.55% for test data in 200 iterations, which indicates the system was successful in distinguishing nitrogen-rich leaves from leaves with a standard content of nitrogen.

## 1. Introduction

In cucumber production, a high consumption of nitrogen occurs more often than a low consumption. Balance in the consumption of nitrogen is very important to reduce nitrate accumulation in green cucumber. Failure to maintain a proper nitrogen ratio disrupts plant metabolism, and nitrogen accumulates in the fruits in the form of nitrate, which reduces the amount of vitamin C by up to 26%, according to Bryk et al. [1].

Excessive consumption of nitrate is harmful to humans, due to the conversion to nitrite by microorganisms in the intestine and stomach.

The demand for high-quality fruits and vegetables has been increasing in the past few decades. Therefore, the mandatory evaluation of food products has become inevitable. Human visual inspection is still widely used, but it is nonetheless subjective, time-consuming, and tedious. The most commonly objective methods are chemical analytical methods such as mass spectrometry (MS) and high-performance liquid chromatography (HPLC). However, they have several disadvantages, including being destructive, time-consuming, and costly. Therefore, accurate, reliable, efficient, and non-destructive options are strongly needed to evaluate the quality-related characteristics of food products (Salimi et al. [2]; Pourdarbani et al. [3]; Sabzi et al. [4]).

The spectroscopy technique does not provide spatial information. On the other hand, computer vision is incapable of inspecting samples of the same color and predicting their chemical components [5]. Thus, by integrating the main advantages of spectroscopy and imaging, the hyperspectral imaging technique can simultaneously obtain spectral and spatial information, which is crucial for predicting the quality of agricultural and food products [6].

Hyperspectral imaging has proven to be an excellent technique to assess the quality of fruits and vegetables and determine contamination, bruising, surface damages, the starch index, firmness, the soluble and solid content, the presence of bitter pit, and cold injury. (Lorente et al. [7]; Leiva-Valenzuela et al. [8]; Cen et al. [9]; Chen et al. [10]). Zhang et al. [11] considered individual wavelengths as independent classifiers and used the receiver performance curve (ROC) to select the best classifiers based on their performance. To develop a low-cost multispectral system for fungal quality control, Esquerre et al. [12] identified wavelengths with the most stable regression coefficients using the Monte Carlo Variable Selection (EMCVS). Mealiness is a negative texture characteristic consisting of abnormal soft tissue with no juiciness in the fruit. Various studies have been done on the detection of mealiness in apples using hyperspectral imaging (Huang & Lu [13]; Huang & Zhu [14]; Huang et al. [15]). Jarolmasjed et al. [16] used hyperspectral imaging to diagnose bitter pit in apples. This method was able to classify apples with an accuracy of 85%. The application of hyperspectral imaging to measure the properties of plums has also been conducted in recent years (Bo et al. [17]). The prediction of firmness in tomato was studied by Yuping et al. [18] using visible and near-infrared spectroscopy. Hyperspectral imaging was used as a powerful tool to identify papaya seeds in black pepper. The results showed that hyperspectral imaging in the NIR region was able to identify black pepper mixed with papaya seeds (Imer et al. [19]). Using hyperspectral imaging, the detection of bursting from the center or hollow heart of potatoes was studied in a non-destructive manner and successfully (Angel et al. [20]. The detection of hollow hearts in potatoes was studied non-destructively using hyperspectral imaging in the range of 1700–10,000 nm. The results revealed that support vector machines (SVM) achieved a correct classification rate of 89.1% (Angel et al. [20]). Williams et al. [21] applied hyperspectral imaging to detect Fusarium damage in maize. Sabzi et al. [22] classified cucumber plants on the basis of their nitrogen content using hybrid ANN-ICA. They concluded that their proposed algorithm was able to early detect nitrogen-rich plants, with a classification rate of 96.11%. Chen et al. [23] studied the early detection of nitrogen in apples using hyperspectral techniques and different methods including support vector machine (SVM), partial least-squares regression (PLSR), random forest (RF), back-propagation artificial neural network (BPANN), and extreme learning machine (ELM). Among these models, nonlinear modeling methods obtained better results than the linear method. The best result was achieved by Rfrog-ELM (R^2^_P_ = 0.843, RMSEP = 2.461 g·kg^−1^, RPD = 2.508).

As mentioned above, the high consumption of nitrogen causes the accumulation of nitrate (NO_3_^−^) in agricultural products. Therefore, scientific management of the use of fertilizers is inevitable to improve the health of consumers by reforming the structure of production. For this purpose, the present study attempted to classify cucumber plants based on consumed nitrogen using hyperspectral imaging and majority voting.

## 2. Results

### 2.1. The Most Effective Wavelengths for the Classification of Cucumber Leaves Based on Nitrogen Content

The wavelengths of 723, 781, and 901 nm were selected as the effective (optimal) wavelengths, and the performance of the classifiers was obtained based on them.

### 2.2. Investigation of the Performance of Different Classifiers for the Classification of Cucumber Leaves Based on Nitrogen Content in 200 Iterations

Table 1 presents the performance of the various classifiers examined in this study using a confusion matrix, the correct classification rate (CCR), and the incorrect classification rate for the test data at 200 iterations. As shown in the table, there are differences between classifiers in the correct classification rate. The highest and the lowest correct classification rates were determined for the ANN-ICA and RBF methods, respectively. Hereupon, to obtain reliable results, it was quite logical to use the unanimity of all classifiers.

The different evaluation criteria of performance for assessing the different classifiers are presented in Table 2. Since the final decision was made by the majority voting method, the results of the MV method are presented in the table. The accuracy of D0 and D2 was the highest, which indicates that the results of both classes are closer to the actual value of the same class. The accuracy was the highest for classes D0 and D2, which means that the results were less different from each other, and the standard deviation of the data was lower. The high sensitivity of classes D0 and D2 indicated that the classifier was more able to correctly distinguish nitrogen-rich leaves. On the other hand, the values of specificity for classes D0 and D2 were higher than those of the other classes, which indicated the ability of the classifier to correctly identify the sample.

The classifier was more able to detect excess nitrogen 48 h (D2) rather than 24 h (D1) after the application of excess N_2_, which is completely reasonable, as the symptoms become more apparent on day D2. It was expected that this trend would continue further on day D3. The failure to meet such an expectation was due to sampling newly grown leaves on day D3.

**Figure 1:**
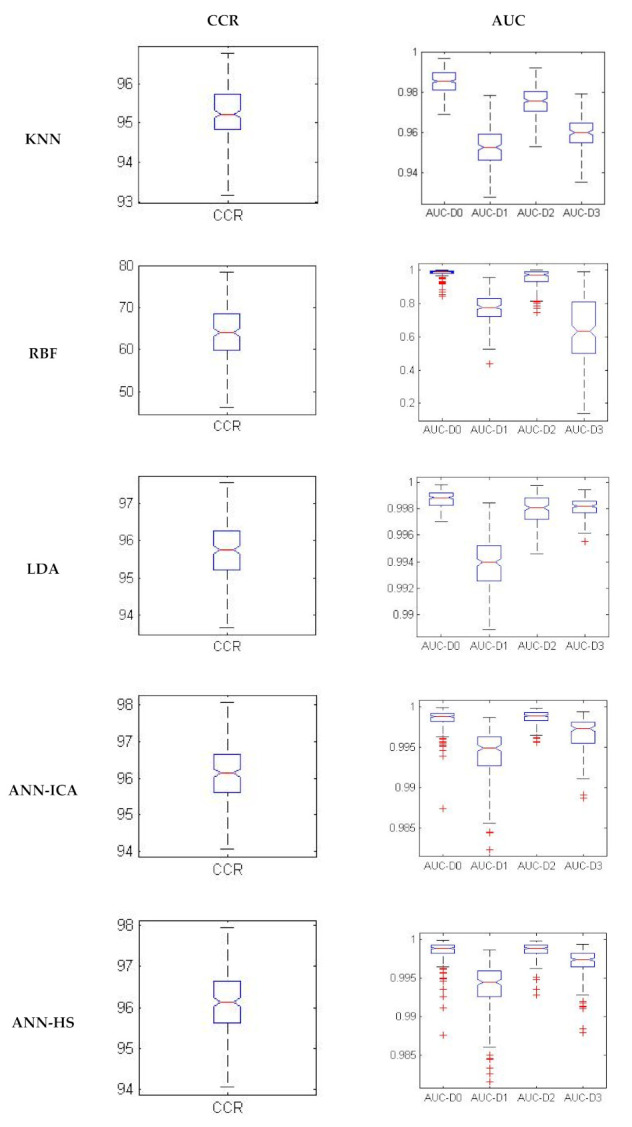

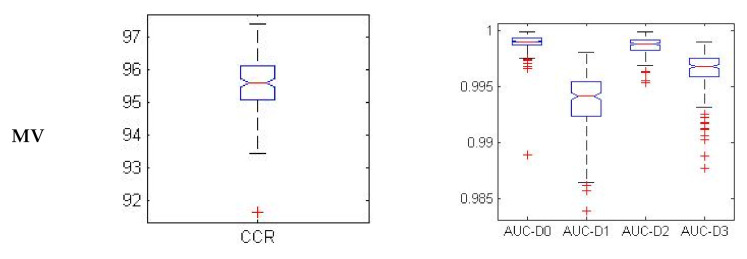
Box plots indicating correct classification rate and area under ROC in 200 repetitions.

Figure 1 evaluates the performance of the classifiers in 200 iterations using box plot of CCR and the area under the ROC curve (AUCs). More compact box plots indicate a higher performance of a classifier. In general, for all classifiers, the box plots of classed D0 and D2 were more compact, which indicated that they identified the excess of nitrogen more accurately.

**Figure 2:**
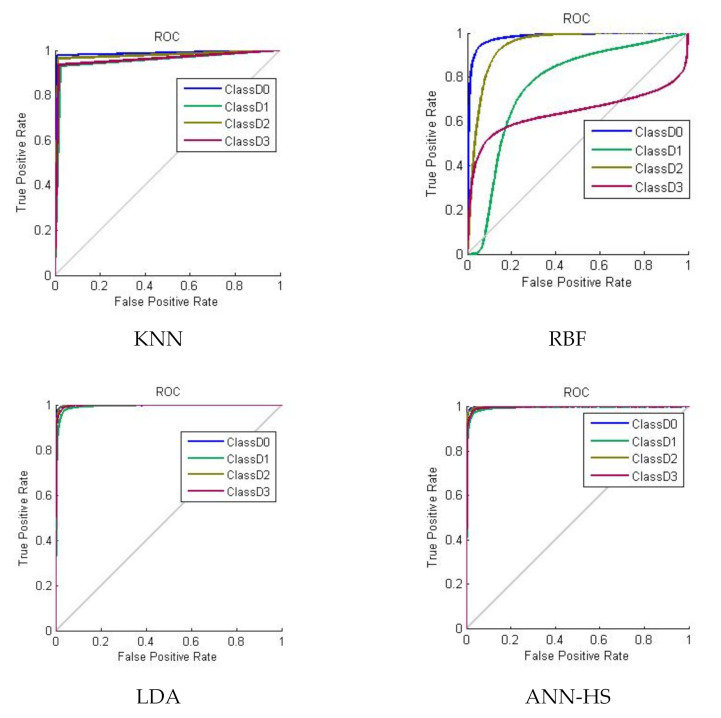

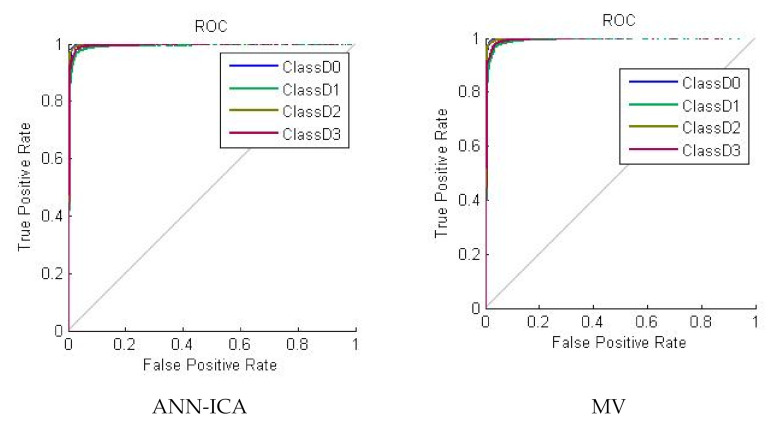
Receiver operating curve of classifiers to classify cucumber leaves in terms of nitrogen content in 200 iterations.

Figure 2 illustrates the receiver operating curve of different classifiers in 200 iterations. A curve farther from the bisector line and closer to the vertical line indicates a high performance of the classifier in that class. Except for the classifier RBF, the others showed a high performance.

## 3. Discussion

### 3.1. Comparison of Mean and Standard Deviation of the ROC and CCR for Effective Wavelength Spectral Data and Entire Data to Identify Cucumber Leaves in Terms of Nitrogen Content

In order to develop a classifier with high speed and low cost, the most effective wavelengths were used to determine the performance of the classifiers. In this section, the results of the classification related to all tested wavelengths and the effective wavelengths are compared. It is obvious that the correct classification rate at the effective wavelength was slightly lower in comparison to that obtained with all wavelengths (Table 3). However, the *t*-test indicated that this difference was not significant (Table 4).

### 3.2. Comparison of the Results Obtained in This Study with Those of Other Researchers

The results obtained in this study were compared with the results of other similar studies. Table 5 shows these comparisons in the form of correct classification rates.

## 4. Materials and Methods

**Figure 3:**
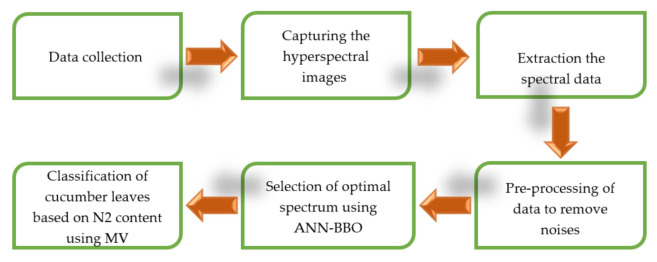
Different stages of the training classification algorithm for cucumber leaves based on nitrogen content.

The different steps performed to classify cucumber leaves based on nitrogen content are shown in Figure 3. As can be seen, the proposed algorithm involves six main stages including data collection, capturing hyperspectral images, extraction of the most effective spectra (optimal wavelength), and classification based on majority voting.

### 4.1. Preparation of the Samples to Perform HyperSpectral Imaginary

**Figure 4:**
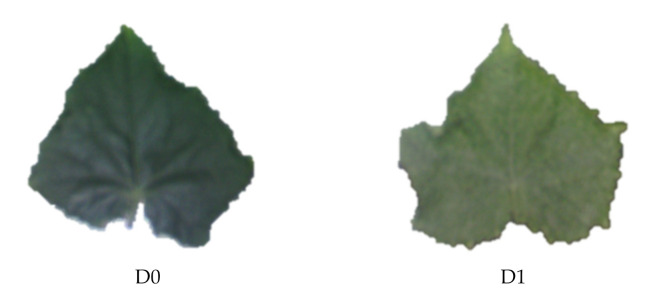

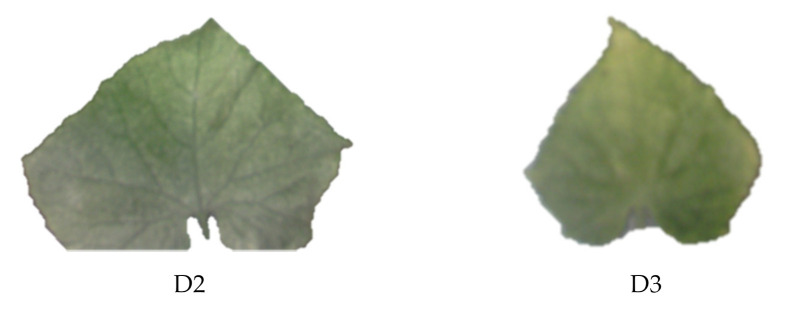
Example of the cucumber leaves on different sampling days.

To prepare the samples including cucumber leaves with standard nitrogen content and excess nitrogen content, cucumber seeds, Super Arshiya’F1 cultivar, was planted in 18 pots (Figure 4). All pots were treated with the same inputs of N_2_ for germination and growth (N_2_ by 2 g/kg of soil). After reaching the appropriate growth, half of the plants received 30% of the excess nitrogen. This amount was measured by precise scales and applied to the soil. Six leaves were picked from each pot on the day before applying excess N_2_ (D0) and 3 consecutive days after applying excess N_2_ (D1 to D3); they were imaged by a hyperspectral camera. The leaves turned pale, and the symptoms of excess N_2_ became quite apparent after 3 days, thus sampling was stopped.

### 4.2. Hardware Required for Classification of Cucumber Plants Based on Nitrogen Content

**Figure 5:**
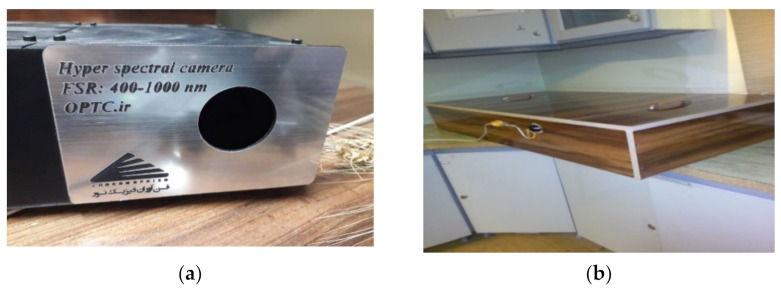
Hardware system for the classification of cucumber plants. (**a**) Super spectral camera, (**b**) lighting enclosure.

In order to obtain hyperspectral images and extract the spectral–spatial properties of images at each individual wavelength, several systems were used, including Labtab (Intel Corei 5, 2430 M at 2.40 GHz, 4 GB of RAM, Windows 10; DELL Co., Round Rock, TX, USA) for data storage and analysis, a hyperspectral camera (made in Fanavaran Physics Co.; Iran-Kashan) (www.optc.ir; accessed on 22 April 2021) with a range from 400 to 1100 nm, two tungsten halogen light sources (SLI-CAL (StellarNet, Tampa, FL, USA)), and a lighting chamber to prevent the ambient light. The camera was located at a horizontal distance of 1 m from the sample, and two light sources were lit on the sample at angle 45°. Figure 5 shows the required hardware.

### 4.3. Preprocessing of Original Spectral Data

The reflectance spectra were converted to absorption spectra for resolving the impact of noise due to ambient light, spectroscopy type, etc. Then, light scattering was corrected by the multiplicative scatter correction (MSC) algorithm. Finally, smoothing was performed by the median filter, using Parles software. (Rossel [28]).

Absorption spectra = log(1/Reflectance spectra)(1)

### 4.4. Selection of the Optimal Wavelength for the Classification of Cucumber Leaves Using Hybrid ANN-BBO

Nowadays, chemical fertilizers are applied as the most economical tool to achieve maximum production per unit area. Major disorders may occur in fruits due to the improper and unbalanced usage of nitrogen. The detection of the nutrient content of leaves is performed through leaf analysis, which is time-consuming and expensive. Thus, to identify nitrogen-rich leaves promptly, it is necessary to develop online algorithms. Undoubtedly, the cost and speed of real-time detection systems are the most important factors. A system of choice achieves the best performance as soon as possible with the least volume of data. Therefore, in this study, the hybrid artificial neural network–biogeography-based optimization (ANN-BBO) algorithm was used to select the most effective wavelengths.

Biogeography is the study of the geographical distribution of living creatures (Simon, [29]). Mathematical simulations of biogeography describe how a species migrates from one habitat to another. Habitats that are more suitable for species have a higher habitat suitability index (HSI). The habitat suitability index depends on factors such as vegetation, rainfall, area, temperature, etc. The variables that determine habitat quality are called suitability index variables (SIVs). In fact, SIVs are independent variables, and HSI is a variable dependent on SIVs. Habitats with high HSIs accommodate more species, and vice versa. On the other hand, habitats with smaller populations show a tendency of the species to migrate more. Maximum migration to a habitat indicates that there are no species in the habitat. As the number of species increases, the habitat becomes more crowded, and fewer species may migrate there.

The method of the hybrid ANN-BBO algorithm is based on introducing different vectors of spectral data in the artificial neural network, and the results of the network are recorded in the form of mean squared error. The output of the network is the class of leaves, determined on the basis of nitrogen content. Any input vector with the least mean square error is considered the optimal vector, and the wavelengths within that vector are known as the optimal wavelengths. Table 6 shows the structure of the hidden layers of the neural network used to select the effective wavelengths.

### 4.5. Measurement of Nitrogen by a Destructive Method

The actual nitrogen content of the leaves was measured by Kjeldahl’s method that includes 3 steps, i.e., digestion, distillation, and titration. For the calculations, we used Equation (2). In this study, the Gerhardt, Kjeldahl (made in German, Königswinter) was used.

(2) $$N\;\mathrm{total}\;(\%)\;=\frac{\;\;\;\left(V_{s}-V_{b}\right)\;\;\;}{md}\;\times \;N_{H2\mathrm{SO}4}\;\times \;0.014_{\mathrm{meqN}}\;\times \;100$$

*V_s_*: Volume consumed by the sample (mL)

*V_b_*: Volume consumed by the control treatment (mL)

N_H2SO4_: Normality of sulfuric acid (eq/L)

*md*: Dry weight of the sample (g)

### 4.6. Classification of Cucumber Plants Based on Nitrogen Content by Majority Voting

First, the performance of different classifiers was investigated, and then the final class was determined based on the majority voting method. The different classifiers used in this study were the hybrid artificial neural network–imperialism competitive algorithm (ANN-ICA), the hybrid artificial neural network–harmonic search (ANN-HS) algorithm, linear discrimination analysis (LDA), the radial basis function network (RBF), and K-nearest-neighborhood (KNN).

#### 4.6.1. Classifier ANN-ICA

The Imperialist Competitive Algorithm is based on artificial intelligence, simulate human communities, and explores the optimal point to resolve the optimization problem (Atashpaz-Gargari & Lucas [30]). This algorithm provides a mathematical model for representing the given problems with a number of random populations called “country”. Some of the best members of the population (elites) are selected as colonizers. Other members are considered a colony. The colonizers attract these colonies towards themselves. The power of an empire is dependent on colonial states and colonies. If an empire fails to succeed in colonial competition, it will be wiped out from the competition. Hence, an empire should attract the colonies of rival empires to guarantee its survival.

In fact, the parameters of research are selected by the imperialist competitive algorithm in the form of vectors and delivered to the artificial neural network. The performance of the network is recorded by the algorithm in the form of squared mean error. The input of the artificial neural network consists of spectral data, and the output is the class of cucumber leaf. Ultimately, the structure with the least mean-squares error is considered the optimal structure.

After the parameters were adjusted optimally, 200 iterations were executed to evaluate the validity of the artificial neural network. For each iteration, 60% of the data were used for training, 30% for testing, and 10% for the validation of the artificial neural network.

#### 4.6.2. Classifier ANN-HS

The harmonic search algorithm was developed according to the process of composing a harmonic piece of music. As known, the gamut of each musical instrument describes the beauty of the song, which means that the gamut of an instrument must be in optimal conditions. Thus, the value of the objective function is determined by the values of the problem variables (Simon et al. [30]). The method for the neural network is the same as the one mentioned above.

#### 4.6.3. Classifier K-Nearest-Neighborhood (KNN)

The k-nearest-neighbor algorithm is often used for classification problems. Implementation of the k-nearest-neighborhood model is possible using the following steps:

(1) Calling the data
(2) Initial selection of k-value
(3) Developing the classes, repeat from 1 to the total number of training data points:
  (A) Calculating the distance of the test data from each row of the training data set by Euclidean distance.
  (B) Selection of the top k rows of the sorted array
  (C) Receiving the most repetitive classes in these rows
  (D) Returning the predicted class value.

#### 4.6.4. Classifier Linear Discrimination Analysis (LDA)

The linear discrimination analysis is performed in three ways: direct, hierarchical and step by step. The step-by-step method is more widely used by researchers because it incorporates independent variables based on predictive power. Therefore, in this study, the stepwise method was used (Anuthama et al. [31]).

#### 4.6.5. Classifier Radial Basis Function (RBF)

An RBF network is a feed-forward network including input layer, hidden layer, and output layer. When the number of iterations or calculated error reaches the desired values, the training of the RBF algorithm is over. A Gaussian function is used as the transfer function. The relationship between the input layer and the hidden layer is expressed using Equation (3), and the relationship between the output layer and the hidden layer is expressed using Equation (4).

(3) $$\omega_{i}\left(x\right)=exp^{\left[-\frac{\|In-C_{i}\|{}^{2}}{2\partial_{i}^{2}}\right](1<i<n)}$$

(4) $$Out_{j}=\overset{n}{\underset{j=1}{\sum }}\beta_{ij}\omega_{i}\left(x\right)(1<j<q)$$

where: *C_i_*, *∂_i_*, and *β_ij_* are the center, width of the hidden layer, and weight between the outputs and the layer, respectively.

### 4.7. Assessment of Performance of the MV Classifier

The performance of the classifier was evaluated by different criteria. These criteria were recall, accuracy, specificity, precision, and F-criteria and graphical criteria of receiver operation curve diagrams (ROC) as well as the area under the ROC curve (Pourdarbani et al. [32]; Alibaba et al. [33]). Table 7 shows the equations.

## 5. Conclusions

The balance of nutrients in the soil is disturbed by fertilizers, and this causes environmental degradation. However, in recent years, farmers have often been excessively using water and fertilizers.

Hyperspectral imaging is a non-destructive and rapid analytical tool for quality assessment of different products and disease diagnosis. In this study, excess nitrogen (by 30%) was added to 18 pots, each containing a plant, that were classified using different classifiers, including the hybrid artificial neural network–imperialism competitive algorithm (ANN-ICA), the hybrid artificial neural network–harmonic search (ANN-HS) algorithm, linear discrimination analysis (LDA), the radial basis function network (RBF), and K-nearest-neighborhood (KNN). Due to differences in the results, the majority voting method was used to obtain a reliable result, since it presents a result based on unanimity.

The wavelengths of 723, 781, and 901 nm were selected as effective wavelengths using the hybrid artificial neural network–biogeography-based optimization (ANN-BBO) algorithm. Then, the performance of the majority voting classifier was evaluated using confusion matrix of CCR and mis-classification rate in 200 iterations. The results revealed that the CCR of the algorithm was 95.55%, indicating good performance in detecting excess nitrogen in cucumber plants.

The early detection of excess fertilizer can improve potting soil or farm soil. One of the ways to improve soil is to use mulching. The mulch layer on nitrogen-rich soil decomposes large amounts of soil nitrogen.

## Figures and Tables

**Table 1:** Performance of different classifiers for the classification of cucumber leaves based on nitrogen content in 200 iterations.

Methods	Classes	D0	D1	D2	D3	Total Data	Misclassified (%)	CCR.
KNN	D0	44,872	948	0	0	45,820	2.11	95.24
	D1	1013	40,091	768	1229	43,101	7.5	
	D2	0	430	29,490	695	30,615	3.81	
	D3	0	1519	773	33,172	35,464	6.9	
RBF	D0	24,241	16,740	2950	1889	45,820	89.01	64.03
	D1	169	36,072	4155	2705	43,101	19.48	
	D2	0	3168	26,837	610	30,615	14.07	
	D3	14	17,809	5543	12,098	35,464	193.13	
LDA	D0	43,703	1751	366	0	45,820	4.84	95.73
	D1	673	40,845	266	1317	43,101	5.52	
	D2	0	395	29,744	476	30,615	2.92	
	D3	0	458	904	34,102	35,464	3.99	
ANN-ICA	D0	44,794	997	29	0	45,820	2.29	96.14
	D1	1087	40,926	133	955	43,101	5.31	
	D2	0	610	29,596	409	30,615	3.44	
	D3	0	915	846	33,703	35,464	5.22	
ANN-HS	D0	44,872	899	49	0	45,820	2.11	96.11
	D1	1079	40,914	151	957	43,101	5.34	
	D2	1	617	29,581	416	30,615	3.49	
	D3	0	1008	847	33,609	35,464	5.51	
MV	D0	44,566	1157	96	1	45,820	2.81	95.55
	D1	1094	40,965	132	910	43,101	5.21	
	D2	8	714	29,486	407	30,615	3.82	
	D3	0	1580	788	33,096	35,464	7.15	

**Table 2:** Evaluation criteria for comparing different classifiers for the classification of cucumber leaves based on nitrogen content in 200 iterations.

Methods	Classes	Recall (%)	Accuracy (%)	Specificity (%)	Precision (%)	F (%)
KNN	D0	97.79231	98.68905	99.08583	97.93103	97.86162
	D1	93.26091	96.15259	97.2771	93.0164	93.1385
	D2	95.034	98.22611	99.05668	96.32533	95.67531
	D3	94.51789	97.22341	98.03675	93.53711	94.02494
RBF	D0	99.25074	82.01636	77.65825	52.90485	69.01942
	D1	48.88534	68.92509	89.98789	83.69179	61.71957
	D2	67.96758	85.79975	95.04128	87.65964	76.56776
	D3	69.92255	77.64791	78.85736	34.11347	45.85529
LDA	D0	98.48341	98.15457	98.01794	95.37975	96.90674
	D1	94.00677	96.82879	97.94545	94.76578	94.38475
	D2	95.08951	98.40386	99.27126	97.15499	96.11116
	D3	95.00488	97.91817	98.82235	96.15949	95.57869
ANN-ICA	D0	97.63083	98.60188	99.02519	97.7608	97.69577
	D1	94.19536	96.94436	98.02753	94.95371	94.57302
	D2	96.70631	98.65802	99.15395	96.67157	96.68894
	D3	96.1103	97.94602	98.49586	95.0344	95.56932
ANN-HS	D0	97.64972	98.65699	99.09759	97.93103	97.79018
	D1	94.18942	96.93468	98.01631	94.92587	94.55621
	D2	96.58156	98.62237	99.1414	96.62257	96.60206
	D3	96.07512	97.87916	98.41753	94.76934	95.41777
MV	D0	97.58693	98.43423	98.80345	97.2632	97.4248
	D1	92.23028	96.365	98.04546	95.0442	93.6161
	D2	96.66907	98.57246	99.05725	96.31227	96.49034
	D3	96.17016	97.57179	97.98271	93.32281	94.72509

**Table 3:** Comparison of means and standard deviations of the AUC and CCR related to effective and all (entire) wavelengths to identify nitrogen-rich leaves in cucumber.

MV Method		CCR	AUC_D0_	AUC_D1_	AUC_D2_	AUC_D3_
Effective wavelength	Mean	95.55	0.998	0.993	0.996	0.995
	SD	0.7725	0.0009	0.0023	0.0007	0.0017
Entire wavelength	Mean	96.14	0.998	0.994	0.998	0.996
	SD	0.0705	0.0013	0.0029	0.0008	0.00020

**Table 4:** Comparison of the correct classification rates related to entire and effective wavelengths by *t*-test.

Statistical Charactreistic	Value
Mean	−1.180 × 10^−1^
Std. Deviation	0.26381678
*t*-Value	−1.001
Degree of Freedom	4
Significance	0.374 ^ns^

^ns^: no significant differences.

**Table 5:** Comparison of the correct classification rates for entire and effective wavelengths by *t*-test.

Researchers	Product Type	Early Detection Type	CCR (%)
Proposed method	Cucumber	Excess nitrogen	95.55
(Xie et al. [24])	Tomato	Gray mold disease	94.44
(Xia et al. [25])	oilseed rape	waterlogging stress	94.44
(Zhang et al. [26])	Tomato	Stress	90
(Xing. & Baerdemaeker [27])	Apple	Bruise	93

**Table 6:** The structure of the hidden layers of the neural network used to select optimal wavelengths.

Parameter	Spesification
Number of Neurons	18 & 16
Number of Layers	2
Transfer Function	poslin, softmax
Back Propagation Network Training Function	trainbr
Back Propagation Weight/Bias Learning Function	learnp

**Table 7:** Equations of performance of MV classifier.

Equations	Description
$\mathrm{Recall}=\frac{TP}{TP+FN}\times 100$	How many samples are correctly detected
$Precision=\frac{TP}{TP+FP}\times 100$	How many correctly detected outputs are actually correct
$\mathrm{Accuracy}\;=\frac{TP+TN}{TP+FN+FP+TN}\times 100$	Total correct classification
$\mathrm{Specificity}\;=\frac{TN}{TN+FP}\times 100$	How many samples are incorrectly detected
$F\_\mathrm{measure}=\frac{2\times \mathrm{Recall}\times Precision}{\mathrm{Recall}+Precision}$	Recall and precision harmonic weighted average
$FP\;Rate=\frac{FP}{TN+FP}\times 100$	Receiver operational curve (ROC)
*TP*: True Positive	*FP*: False Positive
*TN*: True Negative	*FN*: False Negative

## Data Availability

Data sharing not applicable.
